# Restoration of CMV-Specific-CD4 T Cells with ART Occurs Early and Is Greater in Those with More Advanced Immunodeficiency

**DOI:** 10.1371/journal.pone.0077479

**Published:** 2013-10-10

**Authors:** Denise C. Hsu, Stephen J. Kerr, Thatri Iampornsin, Sarah L. Pett, Anchalee Avihingsanon, Parawee Thongpaeng, John J. Zaunders, Sasiwimol Ubolyam, Jintanat Ananworanich, Anthony D. Kelleher, David A. Cooper

**Affiliations:** 1 The Kirby Institute for Infection and Immunity in Society, University of New South Wales, Sydney, Australia; 2 HIV Netherlands Australia Thailand Research Collaboration, Thai Red Cross AIDS Research Centre, Bangkok, Thailand; 3 St Vincent’s Centre for Applied Medical Research, Sydney, Australia; Karolinska Institutet, Sweden

## Abstract

**Objectives:**

Restoration of Cytomegalovirus-specific-CD4 T cell (CMV-Sp-CD4) responses partly accounts for the reduction of CMV-disease with antiretroviral-therapy (ART), but CMV-Sp-CD4 may also drive immune activation and immunosenescence. This study characterized the dynamics of CMV-Sp-CD4 after ART initiation and explored associations with CD4 T cell recovery as well as frequency of naïve CD4 T cells at week 96.

**Methods:**

Fifty HIV-infected, ART-naïve Thai adults with CD4 T cell count ≤350cells/µL and starting ART were evaluated over 96 weeks (ClinicalTrials.gov identifier NCT01296373). CMV-Sp-CD4 was detected by co-expression of CD25/CD134 by flow cytometry after CMV-antigen stimulation.

**Results:**

All subjects were CMV sero-positive, 4 had quantifiable CMV-DNA (range 2.3-3.9 log_10_ copies/mL) at baseline but none had clinically apparent CMV-disease. Baseline CMV-Sp-CD4 response was positive in 40 subjects. Those with CD4 T cell count <100cells/µL were less likely to have positive baseline CMV-Sp-CD4 response (P=0.003). Positive baseline CMV-Sp-CD4 response was associated with reduced odds of quantifiable CMV-DNA (P=0.022). Mean CD4 T cell increase at week 96 was 213 cells/µL. This was associated positively with baseline HIV-VL (P=0.001) and negatively with age (P=0.003). The frequency of CMV-Sp-CD4 increased at week 4 (P=0.008), then declined. Those with lower baseline CMV-Sp-CD4 (P=0.009) or CDC category C (P<0.001) had greater increases in CMV-Sp-CD4 at week 4. At week 96, CD4 T cell count was positively (P<0.001) and the frequency of CMV-Sp-CD4 was negatively (P=0.001) associated with the percentage of naïve CD4 T cells.

**Conclusions:**

Increases in CMV-Sp-CD4 with ART occurred early and were greater in those with more advanced immunodeficiency. The frequency of CMV-Sp-CD4 was associated with reduced naïve CD4 T cells, a marker associated with immunosenescence.

## Introduction

CMV seroprevalence in the population is high, over 90% in Thailand [1]. However, CMV does not generally cause disease unless there is advanced immunodeficiency, such as in advanced HIV-infection [2-4] and in transplant patients [5,6]. 

CMV-Specific (Sp)-CD8 and CD4 T cells are crucial in the control of CMV-infection. In the settings of immunodeficiency secondary to solid organ or stem cell transplant, the presence of CMV-Sp-CD8 T cells [7-9] and CMV-Sp-CD4 T cells [9-13] are associated with lower levels of CMV viraemia and reduced risk of symptomatic CMV disease.

Studies involving recipients of haematopoetic stem cell transplant demonstrated that the adoptive transfer of CMV-Sp-T cells leads to large reductions or even clearance of CMV viraemia [14-16]. However, in those with deficient CMV-Sp-CD4 T cells, the cytotoxic activity of CMV-Sp-CD8 T cells declined after transfer [14]. Thus, CMV-Sp-CD4 T cell help is required for optimal CMV-Sp-CD8 T cell function. 

Antibodies against CMV also play a protective role and are associated with reduced severe sequelae in infants with congenital CMV-infection [17]. In addition, NK cells are also important, demonstrated by the severe manifestation of CMV disease in a patient with a rare NK cell defect [18].

In HIV-negative, CMV sero-positive adults, up to 5% of circulating CD4 T cells are CMV specific [19]. In HIV-infected persons, the proportion of CMV-Sp cells within CD4 T cells can be higher than healthy controls [20,21]. This maybe because large proportions of CMV-Sp-CD4 T cells are also CD57+ [20,22] and are less likely to be infected by HIV [23]. However, in advanced HIV-infection, CMV-Sp-CD4 T cells are more likely to be absent in those with lower CD4 T cell count, especially with a CD4 T cell count of <50 cells/µL [24,25]. 

The presence of CMV-Sp-CD4 T cells is important in HIV-infected persons as it is also associated with protection from CMV viraemia and a lower risk of CMV end organ disease [26]; whereas reduced levels of CMV-Sp-CD4 T cells have been identified in those with CMV disease [25,27-30]. 

Though CMV was a major cause of morbidity and mortality early in the AIDS epidemic [31], the use of antiretroviral therapy (ART) has led to dramatic reductions in the incidence of CMV retinitis [32-34], of up to 80% in some studies [35]. Immune reconstitution resulting from ART also leads to long lasting disease remission [36]. 

The effect of ART on CMV-Sp-CD4 T cells has not been fully elucidated. The majority of published studies were cross-sectional in design [25,37-39] and those that were longitudinal had widely spaced visit intervals, some over years [24,40]. Comparisons of CMV-Sp-CD4 T cell frequency were made between subgroups with different CMV disease status or across widely different CD4 T cell counts. Prospective, longitudinal studies with large number of subjects and frequent monitoring of CMV-Sp-CD4 T cells early after ART initiation are lacking.

There is substantial scientific interest in interventions modifying chronic immune activation in HIV-Infected subjects on suppressive ART [41,42]. Asymptomatic CMV-infection has been associated with immune activation in HIV-infected subjects [43,44]. CMV-Sp-CD4 T cells synthesize type-1 cytokines [45], causing a systemic inflammatory response that is sustained even during latent infection [46]. CMV-Sp-CD4 T cells have been associated with lower CD4 T cell recovery and reduced naïve T cells on ART [47], as well as atherogenesis [48,49]. Recently, Hunt et al have shown reduction in immune activation with the use of Valganciclovir to reduce CMV replication [50]. Therefore, longitudinal data on CMV-Sp-CD4 T cells after ART initiation will likely aid the planning of future interventional studies targeting CMV-Sp-CD4 T cells.

In this study, we explored the dynamics of CMV-Sp-CD4 T cells in 50 subjects with advanced HIV-infection prior to, and longitudinally for 2 years after ART initiation. We also examined factors associated with the presence of CMV-Sp-CD4 T cell response and CMV viraemia prior to the commencement of ART and the associations between CMV-Sp-CD4 T cells, CD4 T cells restoration and immunosenescence. 

## Methods

The study was approved by the Chulalongkorn University Institutional Review Board (Bangkok, Thailand) and Human Research and Ethics Committee of the University of New South Wales (Sydney, Australia) prior to commencement. Written informed consent was obtained from all participants.

### Subjects

Participants were recruited from September 2010 to January 2011 as part of the RESTORE study (ClinicalTrials.gov identifier NCT01296373). The RESTORE study is a prospective observational study that aimed to investigate the process of immune restoration. 

Participants were HIV-infected adults who were treatment naïve, had a CD4 T cell count ≤350 cells/μL and were starting ART. Subjects were reviewed at Baseline (BL), at which time clinical data including age, ethnicity, gender, mode of HIV acquisition, date of diagnosis (estimated/ actual) of HIV-infection, nadir CD4 T cell count, stage of HIV-infection, concomitant medical conditions and medications, BCG vaccination history and history of latent and or active tuberculosis (TB) were collected. Blood was collected for hepatitis B surface antigen (HBsAg), hepatitis C (HCV) and CMV serology, QuantiFERON-TB Gold In-Tube assay (QFN-GIT, Cellestis) and CMV-DNA testing (Roche Diagnostics). Blood was also collected for routine blood tests including CD4 T cell count, HIV viral load (HIV-VL) (The COBAS® AmpliPrep/ COBAS® TaqMan® HIV-1 Test); for evaluation of CMV-Sp-CD4 T cells and for storage. 

All subjects commenced ART consisting of efavirenz, tenofovir and lamivudine at BL. Blood was collected for routine tests, for evaluation of CMV-Sp-CD4 T cells and for storage at each subsequent visit at weeks (wk) 4, 8, 12, 24, 48 and 96. 

### Detection of CMV-Sp-CD4 T cells

CMV-Sp-CD4 T cells were detected using the CD25/CD134 co-expression assay. This assay measures the co-expression of CD25 (α chain of IL-2 receptor) and CD134 (a co-stimulatory molecule that is part of the Tumor necrosis factor receptor superfamily) by CD4 T cells after stimulation with antigens. Zaunders et al has described the methods of detecting antigen specific CD4+ memory T cells using this assay [51]. The assay has also been used to detect Hepatitis-C-specific-CD4 T cells [52]. 

In brief, blood was collected from subjects in Sodium Heparin Tubes (BD Biosciences). Whole blood at 250uL, Iscove’s Modified Dulbecco’s Medium (IMDM; Invitrogen) at 250uL and CMV purified grade III antigen (Meridian Life Sciences) at 4ug/mL were added to 24 well culture plates (BD Biosciences). A positive control consisting of whole blood, IMDM and Leucoagglutinin PHA-L at 2.5ug/mL (Sigma- Aldrich) as well as a negative control (whole blood and IMDM only) were also set up. Culture plates were incubated at 37°C with 5% CO_2_ for 40–48 hrs.

Cells were then stained with CD4-PerCP, CD25-APC, CD134-PE (BD Biosciences) and processed for acquisition on a 2-laser FACSCalibur flow cytometer (BD Biosciences) using Cell Quest Pro software.

**Figure 1:**
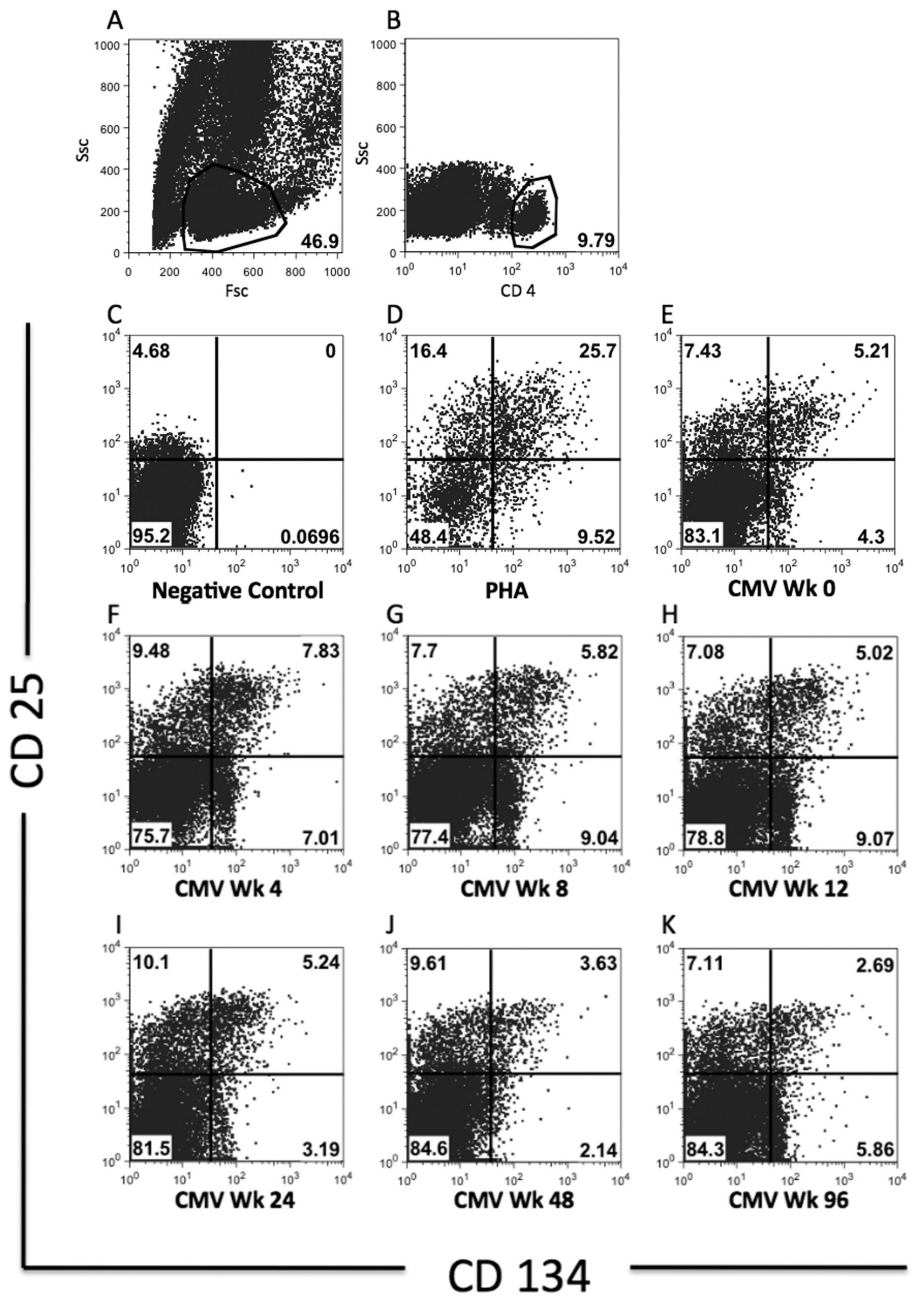
Gating strategies for CMV-SP-CD4 T cells. All flow cytometry plots are from a single subject of the RESTORE study. Lymphocytes were identified using forward and side scatter (Figure 1A), followed by gating on CD4+ T cells (Figure 1B). Gates for CD25+ and CD134+ cells were placed based on comparison with negative control (Figure 1C) and PHA positive control (Figure 1D) to include cells highly co-expressing CD25 and CD134. A representative example of the dynamics of responses to CMV antigens over 96 weeks of follow-up in a RESTORE subject (Figure 1E-K).

Lymphocytes were identified using forward and side-scatter. CD4 T cells were identified using bright CD4 staining. CD25+ and CD134+ cells were gated based on comparison with the negative and positive controls (Figure 1). Positive CMV-Sp-CD4 T cell response was defined as CMV - negative control of ≥0.2% CD4 T cells (mean + 3SD of values from 200 nil antigen tubes); with at least 20 events. Results below the positive cut off were assigned the value of “0”. This was done because results with event count of <20 could not be interpreted with confidence. In addition, variations in the frequency of Ag-Sp-CD4 T cells below the mean+3SD value of negative controls are of questionable significance.

In this study, the CD25/CD134 co-expression assay was modified from that described by Zaunders et al [51] in that CD3 staining was omitted. Others have demonstrated good agreement between CD4+/ side-scatter gating and CD3+CD4+ gating in unstimulated cells [53,54]. Though monocytes also express CD4, they generally have higher side-scatter and lower CD4 intensity when compared to CD4 T cells [53]. We evaluated this approach in this assay prior to commencing this work. We compared 260 data pairs which demonstrated a very good correlation between the 2 different gating strategies in the quantification of CD4 T cell percentage (Spearman rho= 0.997, 95% CI [0.996 – 0.998], P<0.001) as well as the quantification of CD25/CD134 co-expressing cells as a percentage of CD4 T cells (Spearman rho= 0.953, 95% CI [0.940 – 0.963], P<0.001) (Figure S1). Furthermore, the usefulness of this approach of using CD4+/CD25+/CD134+ for identification of Tuberuculosis-Sp-CD4 in comparison with tuberculin skin test and QFN-GIT has previously been published [55].

### Evaluation of Naïve CD4 T cells

Naïve CD4 T cells were identified using flow cytometry. Fresh whole blood was stained with CD3-PerCP, CD4-APC, CD62L-FITC (BD Biosciences) and CD45 RA-PE (BD Pharmingen). Naïve CD4 T cells were defined as CD3+CD4+CD45RA+ and CD62L+.

### Statistical analysis

Statistical analysis was performed with STATA Version 12 (Stata Corporation, College Station, Texas, USA). 

CMV-Sp-CD4 T cell response at baseline was analysed as a categorical variable based on whether the response was positive or not as we were interested in factors associated with the presence of CMV-Sp-CD4 T cell response rather than the magnitude of response. Logistic regression analysis was used to assess characteristics that were associated with presence of CMV-Sp-CD4 T cell response and quantifiable CMV-DNA at baseline. Linearity of continuous covariates was assessed, and in the case of non-linearity the covariate was grouped into quartiles. Adjacent quartile categories were collapsed together if the odds ratio (OR) and size of the confidence intervals (CI) were similar. 

Linear regression was used to explore factors associated with changes in CMV-Sp-CD4 T cell frequency between baseline and wk 4, factors affecting CD4 T cell restoration at wk 96 and factors associated with naïve CD4 T cells at wk 96. 

In the above analyses, factors such as subject demographic characteristics, mode of HIV acquisition, CDC category, history or presence of active or latent tuberculosis, smoking status as well as CD4 T cell count, CD8 T cell count and HIV-VL were screened in univariate models. 

Covariates found to be significant at P≤0.2 in univariate models were included in a multivariate model.  The final model was derived using a forwards stepwise modeling procedure where covariates were added to the model in an iterative manner, in the order of most to least significant P value. Covariates with P>0.05 in the multivariate model were rejected.

McNemar’s test of paired proportions was used to compare the number of subjects with positive CMV-Sp-CD4 T cell response at baseline to subsequent visits. A random effects regression model was used to examine changes in CMV-Sp-CD4 T cell frequency over 96 wks after ART initiation.

## Results

### Baseline characteristics

Fifty Thai participants were recruited. Baseline characteristics have been listed in Table 1. The median age was 32 years (interquartile range, IQR 26-38), 39 subjects (78%) were male. The most common modes of HIV acquisition were male-to-male sex (MSM) and heterosexual transmission, in 34 (68%) and 13 (26%) subjects respectively. 

**Table 1:** Baseline characteristics of the participants in the RESTORE study.

Participants (n)		50
Male (%)		39 (78)
Median age in years (IQR)		32 (26-38)
Mode of HIV acquisition (%)		
	MSM	34 (68)
	Heterosexual	13 (26)
	IVDU	2 (4)
	Others	1 (2)
CDC Category (%)		
	A	27 (54)
	B	15 (30)
	C	8 (16)
Active Hep B (%)		2 (4%)
Chronic Hep C (%)		1 (2%)
TB status		
	Previous Latent	3 (6)
	Previous Active	3 (6)
	New Latent	4 (8)
	New Active	2 (4)
Median baseline CD4 T cell count (cells/µL) (IQR)		186 (113-264)
Baseline CD4 T cell count	<100 cells/µL	11
	100- 199 cells/µL	17
	200- 299 cells/µL	17
	300- 350 cells/µL	5
Median baseline HIV-VL (log_10_ copies/ml) (IQR)		4.9 (4.3-5.3)
Current Smoker (%)		12 (24%)

At baseline, 8 (16%) subjects were CDC category C. All subjects were naïve to any ART except 1 who had prior brief nevirapine exposure >1 year before enrollment. 

Median CD4 T cell count and HIV-VL were 186 cells/µL (IQR 113–264) and 4.9 log_10_ copies/mL (IQR 4.3–5.3) respectively. Three subjects were viral hepatitis co-infected, 2 HBsAg positive and 1 HCV-RNA positive. All subjects were CMV sero-positive and had detectable CMV-DNA (the lower limit of quantification of this assay is 150 copies/mL). However, only 4 subjects had quantifiable CMV-DNA, range 2.3–3.9 log_10_ copies/mL. None had clinically apparent CMV disease. 

### Baseline CMV-Sp-CD4 T cell response was less likely to be positive in those with CD4 count <100 cells/µL

Baseline CMV-Sp-CD4 T cell response was positive in 40 (80%) subjects. Mean (Standard deviation, SD) BL CMV-Sp-CD4 was 2.8 (± 2.6) % of CD4 T cells. In subjects with BL CD4+ T cell <100 cells/µL, 5/11 had positive BL CMV-Sp-CD4 T cell response. Univariate analysis showed that subjects with BL CD4 T cell count <100 cells/µL or CDC category C were less likely to have positive BL CMV-Sp-CD4 T cell response (OR 0.10, 95%CI [0.02 – 0.46], P=0.003) and (OR 0.17, 95%CI [0.03 – 0.85], P=0.032) respectively. CDC category C was no longer statistically significant when assessed using forward stepwise analysis for inclusion in the multivariate model. Baseline HIV-VL, age, gender, mode of HIV acquisition, presence of TB-infection and smoking was not associated with positive BL CMV-Sp-CD4 T cell response.

No statistically significant linear relationships were detected between the magnitude of the CMV-Sp-CD4 T cell response at baseline and the other covariates.

Exploration of factors associated with quantifiable CMV-DNA at baseline found increased odds in those with BL CD4 T cell count <100 cells/µL (OR 14.25, 95%CI [1.31 – 155.23], P=0.029) whereas those with positive BL CMV-Sp-CD4 T cell response had reduced odds (OR 0.06, 95%CI [0.01 – 0.66], P=0.022) of quantifiable CMV-DNA. Positive BL CMV-Sp-CD4 T cell response was the only factor significantly affecting the odds of having quantifiable BL CMV-DNA after forward stepwise analysis whereas BL CD4 T cell count became insignificant. Baseline HIV-VL, age, gender, mode of HIV acquisition, presence of TB-infection and smoking were not associated.

### Response to ART at 96 weeks

All subjects completed 96 weeks of follow-up except 1 who died secondary to intracerebral haemorrhage at wk 52. No subjects developed clinically apparent CMV-disease or CMV immune restoration disease during follow-up.

All subjects were on ART at week 96. Most subjects, 44/49 had HIV-VL <40 copies/mL at week 96 and the rest had low-level viraemia, with HIV-VL <200 copies/mL. Median CD4 T cell count at week 96 was 338 (IQR 275–505) cells/µL. Median (IQR) of CD4 T cell count, HIV-VL and change in CD4 T cell count compared to baseline for each visit have been listed in Table 2.

**Table 2:** Immunologic and Virologic responses to ART.

	BL	Wk 4	Wk 8	Wk 12	Wk 24	Wk 48	Wk 96
Number of Subjects (n)	50	50	50	50	50	50	49
Median (IQR) CD4 count (cells/µL)	186 (113-263)	240 (176–330)	289 (210–394)	289 (187–374)	291 (216-414)	333 (247-474)	338 (275-505)
Median (IQR) change in CD4 count from BL (cells/µL)		64 (31-96)	109 (63-153)	107 (59-148)	114 (56-185)	142 (91-254)	180 (113-281)
Median (IQR) HIV-VL (log_10_copies/ml)	4.9 (4.3-5.3)	2.6 (2.1-2.8)	2.0 (1.6-2.3)	1.6 (1.6-2.1)	1.6 (1.6-1.6)	1.6 (1.6-1.6)	1.6 (1.6-1.6)
Subjects with HIV-VL <40 copies/ml (n)	0	3	15	25	44	44	44
Positive CMV-Sp-CD4 T cell response (n)	40	49	50	49	48	48	48

The mean (SD) change in CD4 T cell count between wk 96 and wk 0 (i.e. CD4 recovery) was 213 (± 151) cells/µL. In multivariate analyses, factors found to be independently associated with CD4 T cell recovery at 96 wks were baseline HIV-VL and age. With each log_10_ higher HIV-VL at baseline, the change in CD4 T cell count increased by 86 cells/µL (95%CI [37 – 136], P=0.001). With each year of older age at, the change in CD4 T cell count was reduced by 8 cells/µL (95%CI [-12 – -3], P=0.003). Frequency of CMV-Sp-CD4 T cells at BL or wk 96 was not associated with change in CD4 T cell count after 96 wks of ART.

### CMV-Sp-CD4 T cells over 96 weeks of therapy

**Figure 2:**
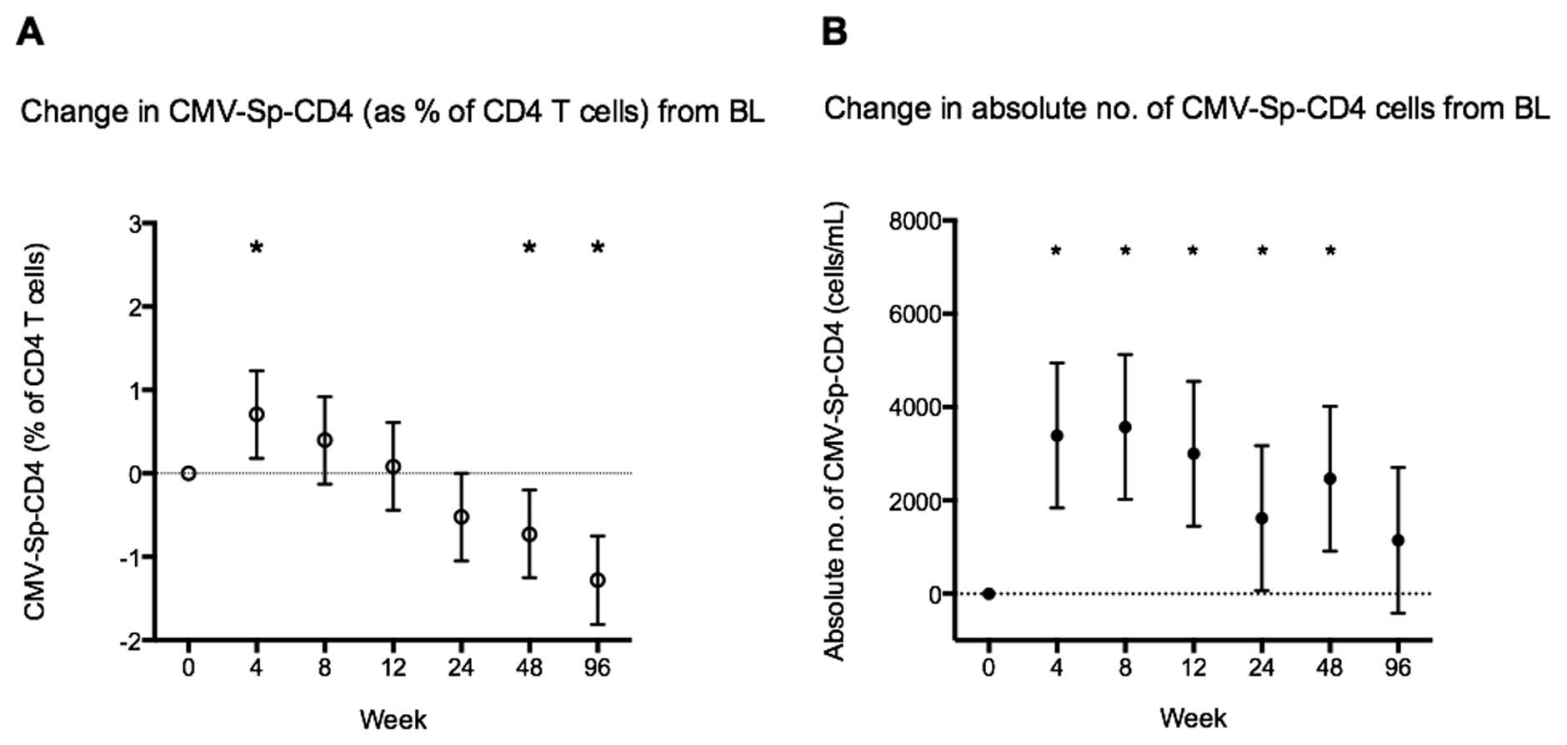
Changes in CMV-Sp-CD4 T cell response. (2a) Mean (95%CI) change in CMV-Sp-CD4 (% of CD4 T cells) when compared to baseline over 96 weeks of ART based on a random effects regression model (* P<0.05). (2B) Mean (95%CI) change in absolute CMV-Sp-CD4 count (cells/mL) when compared to baseline over 96 weeks of ART based on a random effects regression model (* P<0.05).

The number of subjects with positive CMV-Sp-CD4 T cell response increased significantly after 4 wks of ART to 49 (P=0.004) (see Table 2). In a random effects regression model estimating changes in CMV-Sp-CD4 T cell frequency after ART that accounts for within and between subject variability; mean CMV-Sp-CD4 T cells initially increased from 2.8% of CD4 T cell at wk 0 to 3.5% at wk 4 (co-efficient 0.7, 95%CI [0.18 – 1.23], P=0.008), then gradually declined and became significantly lower than baseline at wk 48 (P=0.007) and 96 (P<0.001) (Figure 2a). 

Mean absolute numbers of CD4 T cells responding to CMV also increased significantly after 4 wks of ART, by 3390 cells/mL (95%CI [1840 – 4940], P<0.001) and continued to increase at wk 8, after which it reduced gradually back to levels not significantly different from baseline at week 96 (Figure 2b). 

### Factors associated with initial increase in CMV-Sp-CD4 T cell frequency between wk 0 and 4

Since CMV-Sp-CD4 T cells (as % of CD4 T cells) only increased significantly at wk 4 when compared to wk 0, linear regression was used to explore factors associated with changes in CMV-Sp-CD4 T cell frequency between baseline and wk 4. Univariate analyses found that BL CD4 T cell count (P=0.009) and BL CMV-Sp-CD4 T cell frequency (P=0.024) were negatively associated with changes in CMV-Sp-CD4 T cell frequency between BL and week 4. BL HIV-VL (P=0.036), CDC category C (P<0.001) and change in CD8 T-cell count between BL and week 4 (P=0.014) were positively associated with changes in CMV-Sp-CD4 T cell frequency between baseline and week 4. 

In multivariate analysis, only 2 factors were independently associated with changes in CMV-Sp-CD4 T cell frequency between BL and week 4. CDC category C was positively associated (co-efficient 3.6, 95%CI [1.81 – 5.39], P<0.001) and BL CMV-Sp-CD4 T cell frequency was negatively associated (co-efficient -0.34, 95%CI [-0.60 to -0.09, P=0.009] with change in CMV-Sp-CD4 T cell frequency between BL and week 4.

### CMV-Sp-CD4 T cells and Naïve CD4 cells

Since reduced levels of naïve CD4 T cells have been found to be a marker for immunosenscence and since age and CMV-Sp-CD4 T cell frequency correlated with naïve CD4 cell count [47], we examined the cross sectional relationship between the frequency of naïve CD4 T cells and CMV-Sp-CD4 T cells at wk 96. 

Univariate analysis showed that CD4 T cell count at wk 96 was positively correlated with naïve CD4 T cell percentage whereas CMV-Sp-CD4 T cell frequency at wk 96 and age were inversely correlated with naïve CD4 T cell percentage at wk 96 (Table 3). After fitting variables into multivariate regression analysis using forward stepwise selection, only CD4 T cell count at wk 96 (coefficient per 50 cells higher CD4 T cell count= 2.2, 95%CI [1.5 – 3.0], P<0.001) and CMV-Sp-CD4 T cell frequency at wk 96 (coefficient -5.4, 95%CI [-7.8 – -3.1], P=0.001) remained to be significantly associated with the percentage of naïve CD4 T cells at wk 96. 

**Table 3:** Factors associated with the frequency of naïve CD4 T cells.

	Univariate Analysis		Multivariate Analysis	
Factors	Change co-efficient (95% CI) in naïve CD4 (% of CD4 cells)	P value	Change co-efficient (95% CI) in naïve CD4 (% of CD4 cells)	P value
CD4 count at wk 96 (per 50 cells/µL)	2.2 (1.3 - 3.1)	<0.001	2.2 (1.5 - 3.0)	<0.001
CMV-Sp-CD4 at wk 96 (% of CD4 T cells)	-5.4 (-8.5 - -2.3)	0.001	-5.4 (-7.8 - -3.1)	0.001
Age (yrs)	-0.9 (-1.4 - -0.4)	0.001		
Gender				
Male	5.3 (-4.9 - 15.4)	0.302		
Female	ref			
Mode of HIV acquistion				
MSM	6.9 (-2.1 - 15.9)	0.128		
Others	ref			
CDC category				
CDC A and B	ref			
CDC C	-11.0 (-22.4 - 0.3)	0.057		
TB infection				
Present	-2.5 (-11.9 - 6.8)	0.589		
Absent	ref			
Smoking				
Current smoker	-2.3 (-12.9 - 8.4)	0.672		
Non smoker	ref			

## Discussion

In our cohort of CMV-seropositive subjects with advanced HIV-infection, a majority (80%) had positive CMV-Sp-CD4 T cell responses prior to ART initiation. However, the odds of detecting CMV-Sp-CD4 T cell response were reduced with CD4 T cell count <100 cells/µL. Positive CMV-Sp-CD4 T cell response was associated with reduced likelihood of CMV viraemia. Though this needs to be interpreted with caution given that only 4 subjects had quantifiable CMV viraemia, the above results nonetheless support what has been observed clinically: that HIV-infected subjects generally do not develop clinically apparent CMV disease until CD4 T cell count is <100 cells/µL [4,56] as CMV-Sp-CD4 T cell response is intact and protective at higher CD4 T cell count.

There is a paucity of longitudinal studies documenting the course of CMV-Sp-CD4 T cells with immune restoration after ART. Gerna et al tested CMV lymphoproliferative responses (LPR) at baseline, 3 and 4 years after ART initiation and found that the responses at 3 yrs were greater than baseline but level of responsiveness declined between year 3 and 4 [24]. Keane et al’s retrospective study tested CMV–Sp IFNγ production and found that response increased after the first year of ART but reduced after 3 years of ART. Though most subjects were tested at several time points, serial data was not obtained from the same time points from every subject [40]. The remainder studies in the literature were cross sectional and these found contrasting results. Some found that CMV-Sp-CD4 T cells were lower in HIV-infected subjects treated with ART than in untreated, HIV-infected individuals [38,39], whilst others found that responses were higher than those observed in a HIV-seronegative, CMV-seropositive comparator group [37]. Thus, little is known regarding the dynamics of CMV-Sp-CD4 T cells longitudinally especially during the period early after ART initiation.

We found an early initial increase in the frequency of CMV-Sp-CD4 (as % of CD4 T cells) at week 4, especially in those with advanced immunodeficiency with CDC category C or lower CMV-Sp-CD4 T cells at baseline. In addition, the number of subjects with positive CMV-Sp-CD4 T cell response increased significantly at week 4 after cART initiation and a positive response was detected in all subjects at week 8. 

The absence of positive CMV-Sp-CD4 T cell response in some subjects at baseline maybe due to the low frequency of CMV-Sp-CD4 T cells, below the limit of detection of the CD25/CD134 co-expression assay as a very conservative cut off was used. In addition, CMV-Sp-CD4 T cells might have been present but were not functional. Zhang et al has reported reduced CD134/OX40 expression on CD3 and CD28 stimulated CD4 T cells in HIV-infected subjects when compared with un-infected controls [57].

The initial absence of response was unlikely to be secondary to a complete deletion of all CMV-Sp-CD4 T cell clones given that restoration was seen at week 4. The recovery of CMV-Sp-CD4 T cell responses by *de novo* regeneration of T cells from the thymus would have required a longer period of time since differentiation and emergence of naïve T cells from the thymus alone would take about 4 weeks [58].

The increase in CMV-Sp-CD4 (as % of CD4 T cells) at week 4 maybe due to the redistribution from lymphoid tissues into the peripheral blood secondary to the reduction in viral replication and immune activation after cART initiation [59,60]. Peripheral expansion and differentiation of CMV-Sp-CD4 T cells from naïve CD4 T cells [61-63] also likely contribute to the increase in CMV-Sp-CD4 T cells at week 4. 

After the initial increase, CMV-Sp-CD4 (as % of CD4 T cells) declined gradually. This has also been noted by others [24,40] and maybe secondary to the gradual increase in number and percentage of naïve T cells in the total CD4 T cell pool that occurs with immune recovery [59,64-66]. 

The progressive decline in CMV-Sp-CD4 T cells is likely to be beneficial in the long term as CMV-Sp-CD4 (as % of CD4 T cells) were inversely correlated with the percentage of naïve CD4 T cells. This has also been noted in young adults thymectomized during childhood [67] and in HIV-infected adults [47]. The likely mechanism is that CMV-Sp-CD4 T cells drive ongoing immune activation [46] and differentiation of naïve CD4 T cells into effector or memory phenotypes [68-70].

The negative association between the frequency of CMV-Sp-CD4 T cells and naïve CD4 T cells is a significant finding as decline in naïve T cell levels is part of the phenotype associated with immunosenescence [47,71,72]. In addition, preservation of naïve CD4 T cells is crucial for mounting an immune response to new antigens [73,74]. 

In contrast to Appay et al’s study [47], we did not find an association between the frequency of CMV-Sp-CD4 T cells and the total peripheral blood CD4 T cell count recovery at week 96 post ART initiation. Only baseline HIV-VL and age were significantly associated with CD4 T cell recovery at 96 wks in our study. A possible explanation for this may be that the subjects in Appay et al’s study had much longer duration of ART (3-20 years) [47]. Since CD4 T cell counts continue to increase even after prolonged periods of ART [75,76], subjects in Appay et al’s study thus have more time for immune reconstitution and more time for the effect of CMV-Sp-CD4 T cells on CD4 T cell restoration to manifest.

Recently, Hunt et al have shown that valganciclovir led to suppression of CMV-DNA and reduced T cell activation in HIV-infected subjects on ART. However this trial was too short to observe an effect on CD4 T cell restoration [50]. Our results on the dynamics of CMV-Sp-CD4 T cells post ART initiation may help the selection of subjects who may benefit most from anti-CMV therapy. A larger trial of anti-CMV therapy targeting the subgroup of ART treated HIV-infected subjects with persistently elevated CMV-Sp-CD4 T cells, would provide valuable information on whether this can partly reverse the immunosenescent phenotype associated with HIV-infection. 

There are several potential limitations in this study. Firstly, CMV antibody results used to determine sero-status of the subjects were qualitative but not quantitative. Secondly, CMV PCR was only performed at baseline but not longitudinally. Data from the literature suggest that the majority of subjects with CMV viraemia become CMV PCR negative in the first 3 months post ART initiation and over 70% of patients remain persistently CMV PCR negative [77-79]. Given that only 4 patients had quantifiable CMV viraemia at baseline, the yield of CMV PCR after ART initiation would have been low. Thirdly, though CMV-Sp-CD8 T cell response is important in the control of CMV infection, this has not been assessed as it is beyond the scope of this study. Fourthly, other markers of T cell senescence such as CD27-CD28-CD57+ cells or telomere were not assessed. However, Appay et al have found that reduced naive T cell levels rather than the accumulation of CD57+ senescent T cells are better at identifying the immunosenescence phenotype associated with HIV disease progression [47].

In summary, we found that CD4 T cell count <100 cells/µL was associated with reduced odds of positive CMV-Sp-CD4 T cell response. Positive CMV-Sp-CD4 T cell response was associated with reduced odds of CMV-viraemia. After initiation of ART, there was an early increase in the frequency of CMV-Sp-CD4 T cells followed by a gradual decline. Higher CMV-Sp-CD4 T cell frequency after 96wks of ART was associated with lower percentage of naïve CD4 T cells, one of the markers associated with the immunosenescent phenotype. This knowledge is likely to be important for future studies on reversing the effect of immunosenescence in HIV-infected patients.

## Supporting Information

Figure S1 **Comparison of gating strategies with and without CD3 staining.** Lymphocytes were gated on forward and side scatter (S.1A). CD4 were gated using CD3+CD4+ (S.1B) or CD4+ (S.1D). Frequency of CD25+CD134+ cells based on the 2 respective gating strategies (S.1C, S.1E). Correlations between the 2 gating strategies for quantification of percentage of CD4 T cells (S1.F) and percentage of CD25+CD134+ co-expressing cells (S1.G) based on 260 paired data values.
(TIFF)
