# Supplementary figures and images for: Restoration of CMV-Specific-CD4 T Cells with ART Occurs Early and Is Greater in Those with More Advanced Immunodeficiency
Source: PLoS One. 2013 Oct 10;8(10):e77479. doi: 10.1371/journal.pone.0077479 (PMC3795037; doi:10.1371/journal.pone.0077479)

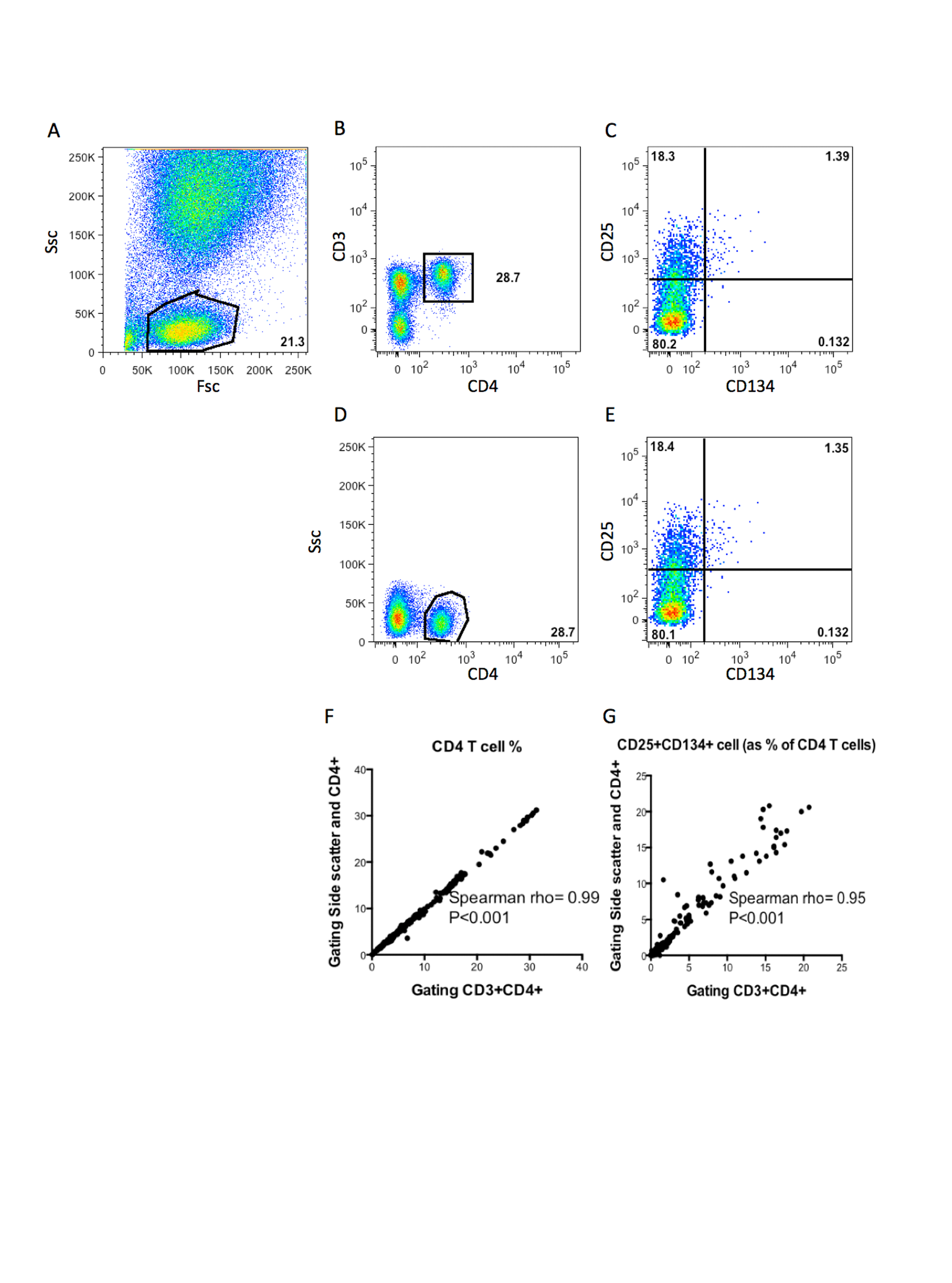

Supplement: Figure S1 — Comparison of gating strategies with and without CD3 staining. Lymphocytes were gated on forward and side scatter (S.1A). CD4 were gated using CD3+CD4+ (S.1B) or CD4+ (S.1D). Frequency of CD25+CD134+ cells based on the 2 respective gating strategies (S.1C, S.1E). Correlations between the 2 gating strategies for quantification of percentage of CD4 T cells (S1.F) and percentage of CD25+CD134+ co-expressing cells (S1.G) based on 260 paired data values. (TIFF) [file pone.0077479.s001.tiff]
